# Risk and Protective Factors for Personality Disorders: An Umbrella Review of Published Meta-Analyses of Case–Control and Cohort Studies

**DOI:** 10.3389/fpsyt.2021.679379

**Published:** 2021-09-06

**Authors:** Marco Solmi, Elena Dragioti, Giovanni Croatto, Joaquim Radua, Stefan Borgwardt, Andre F. Carvalho, Jacopo Demurtas, Anna Mosina, Peter Kurotschka, Trevor Thompson, Samuele Cortese, Jae Il Shin, Paolo Fusar-Poli

**Affiliations:** ^1^Department of Psychiatry, University of Ottawa, Ottawa, ON, Canada; ^2^Department of Mental Health, The Ottawa Hospital, Ottawa, ON, Canada; ^3^Early Psychosis: Interventions and Clinical-Detection (EPIC) Lab, Department of Psychosis Studies, Institute of Psychiatry, Psychology, London, United Kingdom; ^4^Faculty of Environmental and Life Sciences, Center for Innovation in Mental Health, School of Psychology, University of Southampton, Southampton, United Kingdom; ^5^Clinical Epidemiology Program, Ottawa Hospital Research Institute, Ottawa, ON, Canada; ^6^Pain and Rehabilitation Centre, and Department of Health, Medicine and Caring Sciences, Linköping University, Linköping, Sweden; ^7^Neurosciences Department, University of Padua, Padua, Italy; ^8^Imaging of Mood- and Anxiety-Related Disorders (IMARD) Group, Institut d'Investigacions Biomèdiques August Pi i Sunyer (IDIBAPS), CIBERSAM, Barcelona, Spain; ^9^Department of Clinical Neuroscience, Centre for Psychiatric Research and Education, Karolinska Institutet, Solna, Sweden; ^10^Department of Psychiatry, Medical Faculty, University of Basel, Basel, Switzerland; ^11^Department of Psychiatry, Psychosomatics and Psychotherapy, University of Lübeck, Lübeck, Germany; ^12^Department of Psychiatry, Faculty of Medicine, University of Toronto, Toronto, ON, Canada; ^13^Centre for Addiction and Mental Health, Toronto, ON, Canada; ^14^Clinical and Experimental Medicine PhD Program, University of Modena and Reggio Emilia, Modena, Italy; ^15^Clienia AG, Wetzikon Psychiatric Centre, Wetzikon, Switzerland; ^16^Department of General Practice, University Medical Center Würzburg, Würzburg, Germany; ^17^Faculty of Education and Health, University of Greenwich, London, United Kingdom; ^18^Clinical and Experimental Sciences (CNS and Psychiatry), Faculty of Medicine, University of Southampton, Southampton, United Kingdom; ^19^Solent NHS Trust, Southampton, United Kingdom; ^20^Hassenfeld Children's Hospital at NYU Langone, New York University Child Study Center, New York, NY, United States; ^21^Division of Psychiatry and Applied Psychology, School of Medicine, University of Nottingham, Nottingham, United Kingdom; ^22^Department of Pediatrics, Yonsei University College of Medicine, Seoul, South Korea; ^23^Department of Brain and Behavioural Sciences, University of Pavia, Pavia, Italy; ^24^Department of Psychosis Studies, Institute of Psychiatry, Psychology & Neuroscience, King's College London, London, United Kingdom; ^25^Outreach and Support in South London (OASIS) Service, South London and Maudsley NHS Foundation Trust, London, United Kingdom

**Keywords:** umbrella review, personality disorder, prevention, meta-analysis, risk factor, systematic review, psychiatry, mental health

## Abstract

The putative risk/protective factors for several personality disorders remain unclear. The vast majority of published studies has assessed personality characteristics/traits rather than disorders. Thus, the current umbrella review of meta-analyses (MAs) aims to systematically assess risk or protective factors associated with personality disorders. We searched PubMed–MEDLINE/PsycInfo databases, up to August 31, 2020. Quality of MAs was assessed with AMSTAR-2, while the credibility of evidence for each association was assessed through standard quantitative criteria. Out of 571 initial references, five meta-analyses met inclusion criteria, encompassing 56 associations of 26 potential environmental factors for antisocial, dependent, borderline personality disorder, with a median of five studies per association, and median 214 cases per association. Overall, 35 (62.5%) of the associations were nominally significant. Six associations met class II (i.e., highly suggestive) evidence for borderline personality disorder, with large effect sizes involving childhood emotional abuse (OR = 28.15, 95% CI 14.76–53.68), childhood emotional neglect (OR = 22.86, 95% CI 11.55–45.22), childhood any adversities (OR = 14.32, 95% CI 10.80–18.98), childhood physical abuse (OR = 9.30, 95% CI 6.57–13.17), childhood sexual abuse (OR = 7.95, 95% CI 6.21–10.17), and childhood physical neglect (OR = 5.73, 95% CI 3.21–10.21), plus 16 further associations supported by class IV evidence. No risk factor for antisocial or dependent personality disorder was supported by class I, II, and III, but six and seven met class IV evidence, respectively. Quality of included meta-analyses was rated as moderate in two, critically low in three. The large effect sizes found for a broad range of childhood adversities suggest that prevention of personality disorders should target childhood-related risk factors. However, larger cohort studies assessing multidimensional risk factors are needed in the field.

## Introduction

Personality disorders are defined as “an enduring pattern of inner experience and behavior that deviates markedly from the expectations of the individual's culture, is pervasive and inflexible, has an onset in adolescence or early adulthood, is stable over time, and leads to distress or impairment.”

It has been reported that globally, personality disorders have a prevalence around 3 to 10% in the global population (1), and much higher in people affected by other mental disorders (2), and so it is considered a global mental health priority (3). Their peak age at onset is at age 20.5 years (4).

In the last 50 years, a lot of interest went into personality disorders, promoting their status from an unreliable and not so valid diagnosis before the 1960s, to a condition with clear diagnostic criteria in particular after introduction of DSM-III (5). Then, DSM-based criteria have raised criticism, and alternative diagnostic frameworks have been proposed (1). They are now classified differently in Diagnostic and Statistical Manual (DSM), 5th version (6) and in International Classification Diseases (ICD)-11 (7). DSM-V classifies personality disorders in three clusters (A, B, and C) and “other personality disorders.” Cluster A includes paranoid personality disorder, schizoid personality disorder, and schizotypal personality disorder; cluster B includes antisocial personality disorder, borderline personality disorder, histrionic personality disorder, and narcissistic personality disorder; cluster C includes avoidant personality disorder, dependent personality disorder, and obsessive-compulsive personality disorder (6). ICD-11 applies a different approach, with the aim to identify fewer categories, which overlap less and ultimately have greater clinical utility (1). Specifically, ICD-11 categorizes personality disorders (6D10) into mild personality disorder, moderate personality disorder, and severe personality disorder, in addition to which Prominent personality traits or patterns (6D11), namely, Negative affectivity, Detachment, Dissociality, Disinhibition, Anankastic, or Borderline pattern in personality disorder or difficulty, must be further specified (7).

Structural brain alterations, namely, bilateral gray matter reductions in concentrations in ventral cingulate gyrus, medial temporal lobe, and fronto-limbic structures, are associated with different personality disorders defined according to several versions of DSM (8–11). Beyond cross-sectional associations with phenotypes and biomarkers, several risk factors for personality disorders have also been described. An overview of systematic reviews (12) focused on systematic reviews on risk factors for personality disorders. However, it only focused on parenting style, and it also focused on qualitative reviews rather than quantitative meta-analyses (12). Also, evidence from meta-analyses is frequently biased, and the credibility of the claimed associations between putative risk or protective factors for personality disorders remains unknown. To fill this gap in the literature, we conducted an umbrella review focused on environmental risk and protective factors for personality disorders, to identify and measure possible methodological limitations and sources of bias in the published and unpublished evidence, which might have underestimated or inflated claimed associations, as previously shown in several previous umbrella reviews on risk factors for mental disorders or obesity (13–17). Therefore, the aim of this umbrella review was to grade the evidence from meta-analyses of cohort and case–control studies on protective and risk factors for personality disorders accounting for several sources of bias and applying established quantitative criteria.

## Methods

This umbrella review adhered to state-of-the-art methods of previously published or planned umbrella reviews (15, 18–23), and according to the Meta-analysis of Observational Studies in Epidemiology (MOOSE) and the Preferred Reporting Items for Systematic Reviews and Meta-analyses (PRISMA) were followed in conducting and reporting this umbrella review (Supplementary Tables 1, 2) (24, 25). The study followed an *a priori* protocol, available on request. MS, AFC, PF-P designed the study, prepared the search key, and drafted the protocol. ED run the statistical analyses. Four investigators (MS, ED, AM, and PK, all MDs) divided into two couples independently performed literature screening and data extraction, including quality assessment of included meta-analyses.

### Literature Search Strategy

We conducted a systematic search in PubMed and PsycINFO from inception to August 31, 2020. We included meta-analyses of case–control and cohort studies that assessed risk or protective factors for personality disorders, defined according to ICD or DSM, any version (6, 7, 26). The search strategy was “(personality disorder) AND meta-analysis.” No restrictions regarding year of publication, language, country, ethnicity, or any other characteristic were applied during the search process. We also hand searched references of included meta-analyses and other relevant articles. When authors did not agree regarding screening or data extraction, a third author (MS) resolved any conflict, reaching a consensus with the two authors.

### Eligibility Criteria

We only included systematic reviews that also conducted a quantitative meta-analysis pooling data from case–control or cohort (either retrospective or prospective) studies reporting on environmental factors that may affect the risk of the disorders of interest, as per the ICD or DSM criteria. Specifically, we included disorders that corresponded to *ICD-10 “F60 Specific personality disorders”* (26) and “*Personality disorder”* in DSM-5 (6). Risk or protective factors of interest were deemed eligible, regardless of the direction of the association (protective or risk factor). No language restriction was applied.

Meta-analyses of studies that included other-than-human population, having a cross-sectional design, as well as focusing on genome-wide associations or on single nucleotide polymorphism were excluded. Also, systematic reviews without a quantitative meta-analytic data synthesis, narrative reviews, and commentaries/letters to the editor were not included in the present umbrella review.

Finally, if multiple meta-analyses investigated the same risk or protective factor and the same outcome, only the meta-analysis with the largest number of studies pooled to measure the association was retained.

### Data Extraction

We extracted information into a standardized pre-defined template. The list of variables of interest included PMID/DOI of the included study, first author, year of publication, design of included studies (cohort, case–control), number of included studies in the meta-analysis, specific population cohort (i.e., general population, primary school, secondary school, university students, hospital sample, or a sample with a specific somatic, mental, or somatic/mental comorbid condition, etc.) as well as the reference/comparison population (i.e., no risk factor in cohort studies, no disorder in case–control studies), tools for the definition of both population and risk/protective factor (DSM, ICD, clinical records, rating scales), specific protective or risk factor, outcome (ICD or DSM code if available, or definition of specific disorders as reported by authors given inclusion criteria were met), and its risk estimate. We assessed the methodological quality of included meta-analyses as independent couples of two investigators (JD, PK, AM, and MS, all MDs) by means of the AMSTAR (A Measurement Tool to Assess Systematic Reviews) version 2 (27). If needed, we contacted authors to ask for data.

### Data Analysis

For each association (i.e., between each specific risk or protective factor and personality disorder), we pooled effect sizes of individual studies reported in each meta-analysis, as well as cases developing personality disorder and total sample size, and recalculated the pooled effect sizes with its 95% CIs, using random-effects models (28). We transformed the effect sizes or modified the direction of associations reported in original publications only for the associations with continuous or correlational data (e.g., Hedges g, beta coefficients) to present comparable estimates (i.e., equivalent odds ratio—eOR) (22). Heterogeneity was measured with the *I*^2^ statistic (29). In addition, 95% prediction intervals for the effect sizes were computed to estimate the possible range in which the effect sizes of future studies were anticipated to fall (30). We also examined small-study effect bias, testing whether smaller studies generated larger effect sizes compared with larger studies (15, 18–22, 31). Specifically, as indicators of small-study effect, we used both the Egger regression asymmetry test (*p* ≤ 0.10) and whether the random-effects summary effect size were larger than the effect size of the largest study contributing to that association (18, 20, 21, 31). We finally measured the presence of excess significance bias by assessing whether the observed number of studies with nominally statistically significant results was different from the expected number of studies with statistically significant results (32, 33). The expected number of statistically significant studies per association was calculated by summing the statistical power estimates for each component study. The power estimates of each component study depend on the plausible effect size for the examined association, which we assumed to be the effect size of the largest study (i.e., the smallest SE) per association (33). For excess significance bias, a *p* ≤ 0.10 was considered statistically significant (32). All analyses were conducted in Stata/MP, version 10.0 (StataCorp LLC).

### Assessment of the Credibility of Evidence, and Sensitivity Analyses

In line with former umbrella reviews (14, 16, 17, 34). eligible associations for personality disorders were classified into five levels according to the strength of the evidence of potential environmental risk/protective factors: convincing (class I), highly suggestive (class II), suggestive (class III), weak (class IV), and not significant (NS) (Table 1).

**Table 1:** Main criteria for evaluation of the credibility of the evidence of observational studies.

**Classification**	**Criteria**
Convincing evidence (class I)	1. More than 1,000 cases 2. Significant summary associations (*p* < 1 × 10^−6^) per random-effects calculations 3. No evidence of small-study effects 4. No evidence of excess of significance bias 5. Prediction intervals not including the null value 6. Largest study nominally significant (*p* < 0.05) 7. No large heterogeneity (i.e., *I^2^* <50%)
Highly suggestive evidence (class II)	1. More than 1,000 cases 2. Significant summary associations (*p* < 1 × 10^−6^) per random-effects calculation 3. Largest study nominally significant (*p* < 0.05)
Suggestive evidence (class III)	1. More than 1,000 cases 2. Significant summary associations (*p* < 1 × 10^−3^) per random-effects calculations
Weak evidence (class IV)	1. All other associations with *p* ≤ 0.05
Non-significant associations (NS)	1. All associations with *p* > 0.05

Sensitivity analyses were conducted removing the >1,000 cases criterion.

## Results

### Search Results

**Figure 1:**
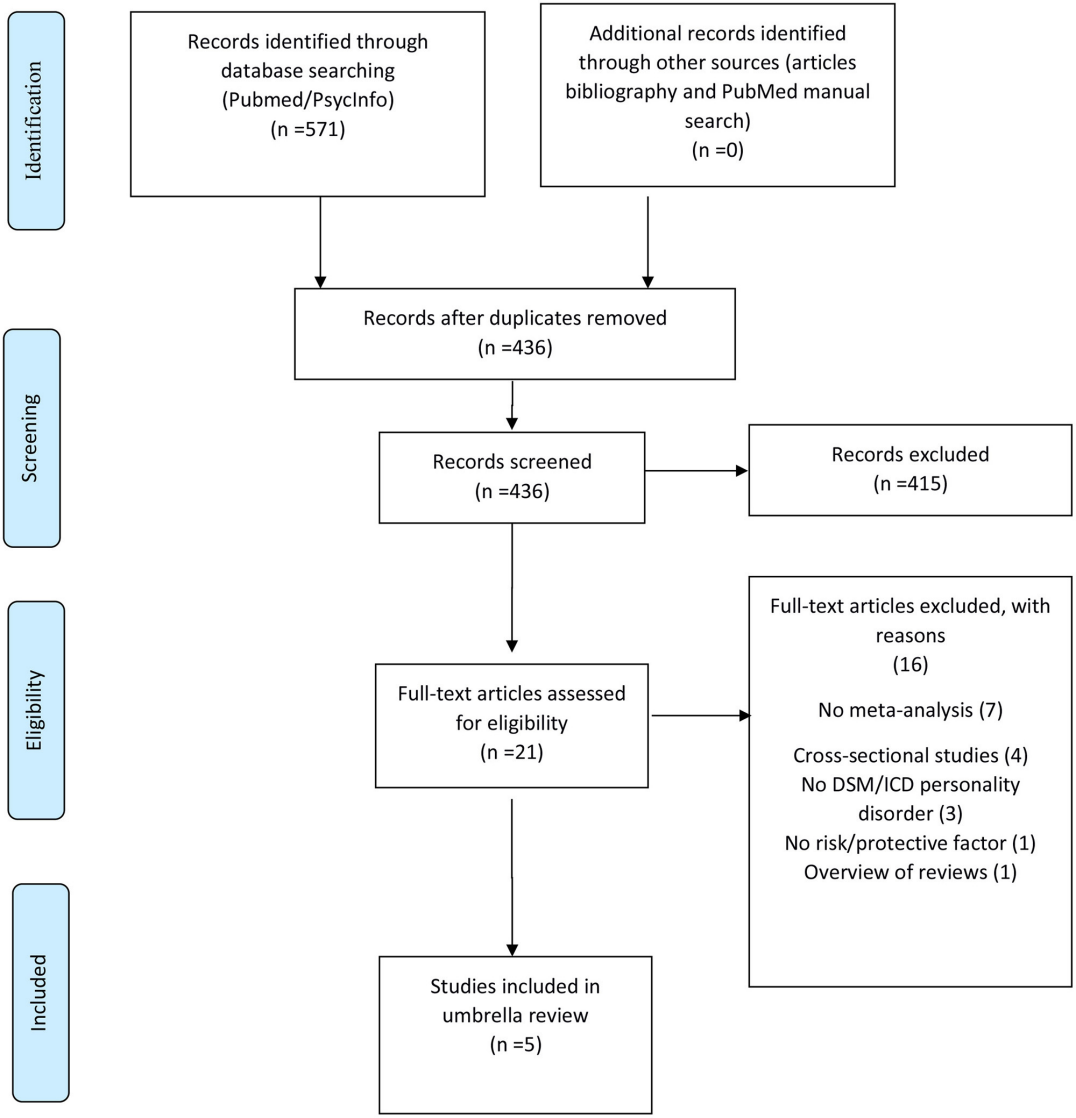
PRISMA flow chart, last search August 31, 2020.

Out of 571 initial hits, 436 were assessed at title and abstract level, and 415 were excluded. The remaining 21 were assessed at full-text level, and 16 were excluded, ultimately including 5 meta-analyses. The list of excluded meta-analyses after full-text assessment, with specific reason for exclusion, is reported in online Supplementary Material. Finally, five meta-analyses were included in this umbrella review (35–39) (Table 2). The study selection flow is reported in Figure 1. The whole dataset with results from individual studies is available on reasonable request.

**Table 2:** Characteristics of eligible articles.

**References**	**Number of associations**	**Potential environmental risk/protective factor**	**Personality disorder**	**AMSTAR 2 quality**
Fossati et al. (35)	21	Sexual abuse (overall; between age 0 and 6; between age 13 and 18; by father; by mother; by sibling; by other relatives; by non-relatives; by any caretaker; with use of force; with disclosure; with help; with fondling; with genital fondling; with oral sex; with penetration; longer duration; higher severity; higher frequency; higher number of perpetrators	Borderline personality disorder	Critically low
Kane and Bornstein (37)	5	Any childhood adversity; physical abuse; sexual abuse; emotional abuse; neglect	Dependent personality disorder	Critically low
Nottell (39)	25	Genetic risk/mental disorder; genetics; temperament; IQ; disorders/pathology during childhood; experiential/external factors; physical abuse; sexual abuse; emotional abuse; separation and loss; poor school; medical incidents; family socioeconomic status; family dysfunction; interpersonal factors; neglect; rejecting; unstable or erratic; inconsistent discipline; harsh parenting; parent delinquency; delinquent sibling; parent disorder/pathology; social desirability; behavioral factors (child antisocial behavior)	Antisocial personality disorder	Critically low
Porter et al. (36)	6	Any childhood adversity; physical abuse; sexual abuse; emotional abuse; emotional neglect; physical neglect	Borderline personality disorder	Moderate
Winsper et al. (38)	5	Sexual abuse; physical abuse; hostility/verbal abuse; neglect; parental conflict	Borderline personality disorder; Borderline personality disorder symptoms	Moderate

### Descriptive Results of the Included Associations

The characteristics of included studies are described in Table 2. The five eligible meta-analyses corresponded to 56 with available data for synthesis, between 26 potential putative risk/protective factors and 3 personality disorders (antisocial, borderline, and dependent personality disorder) (Tables 2, 3). The eligible meta-analyses were published between 1999 and 2020. The median number of studies per association was 5 [interquartile range (IQR) = 3–14]. The median number of cases per association was 214 (IQR = 98–2,420) and the number of cases was >1,000 in 14 associations, while in 2 associations the number of cases was not reported (Tables 3, 4). All eligible meta-analyses used summary-level data from published literature. No protective factors were identified.

**Table 3:** Potential significant environmental risk/protective factors of personality disorders.

**References**	**Risk factor**	**Total number of cases/total population**	**Number of primary studies**	**Effect size metric**	**Random effects summary effect size (95% CI)**	***P* random**	**95% PI (including null value)**	***I^**2**^* %**	**Small-study effects/excess statistical significance**	**eOR**	**Level of evidence**	**Level of evidence 2 (–*n*) > 1,000**
**Antisocial personality disorder**												
Nottell (39)	Genetic risk/mental disorders	154/7,607	10	OR	4.10 (2.28, 2.76)	2.3 × 10^−6^	Yes	89.5	Yes/yes	4.10	IV	III
Nottell (39)	Disorders/pathology during childhood	114/662	5	OR	12.82 (4.68, 35.11)	6.9 × 10^−7^	Yes	55.4	No/no	12.82	IV	II
Nottell (39)	Interpersonal factor	965/4,280	17	OR	1.66 (1.16, 2.37)	0.006	Yes	76.4	No/NP	1.66	IV	IV
Nottell (39)	Rejecting	109/470	2	OR	2.11 (1.36, 3.27)	0.001	NA	0.00	NA/yes	2.11	IV	IV
Nottell (39)	Parent disorder/pathology	537/2,086	9	OR	2.08 (1.26, 3.44)	0.004	Yes	73.4	No/no	2.08	IV	IV
Nottell (39)	Behavioral factor	177/851	4	OR	9.16 (5.96, 14.07)	1.0 × 10^−4^	No	0.0	No/no	9.16	IV	III
**Borderline personality disorder**												
Porter et al. (36)	Any childhood adversity	16,098/182,816	97	OR	14.32 (10.80, 18.98)	1.59 × 10^−76^	No	96.7	Yes/yes	14.32	II	II
Porter et al. (36)	Childhood physical abuse	2,869/37,732	30	OR	9.30 (6.57, 13.17)	2.7 × 10^−36^	No	76.4	Yes/yes	9.30	II	II
Porter et al. (36)	Childhood sexual abuse	3,748/37,756	31	OR	7.95 (6.21, 10.17)	4.54 × 10^−61^	No	67.5	Yes/yes	7.95	II	II
Porter et al. (36)	Childhood emotional abuse	3,525/37,287	27	OR	28.15 (14.76, 53.68)	3.8 × 10^−24^	Yes	94.1	Yes/yes	28.15	II	II
Porter et al. (36)	Childhood emotional neglect	3,225/36,587	21	OR	22.86 (11.55, 45.22)	2.4 × 10^−19^	Yes	94.9	Yes/yes	22.86	II	II
Porter et al. (36)	Childhood physical neglect	3,072/36,449	20	OR	5.73 (3.21, 10.21)	3.4 × 10^−9^	Yes	93.4	No/yes	5.73	II	II
Winsper et al. (38)	Sexual abuse	231/660	9	d	0.70 (0.53, 0.87)	1.1 × 10^−15^	No	0.0	Yes/yes	3.20	IV	IV
Winsper et al. (38)	Physical abuse	290/2,522	5	d	0.56 (0.33, 0.79)	2.2 × 10^−6^	No	0.0	No/yes	2.76	IV	IV
Winsper et al. (38)	Hostility/verbal abuse	110/8,442	5	d	0.62 (0.46, 0.78)	8.9 × 10^−15^	No	11.7	No/NP	3.07	IV	I
Winsper et al. (38)	Neglect	109/268	4	d	0.58 (0.03, 1.13)	0.040	Yes	85.0	Yes/yes	2.86	IV	IV
Fossati et al. (35)	Sexual abuse, overall	705/2,260	19	OR	4.43 (3.43, 5.73)	1.0 × 10^−5^	No	0.0	Yes/NP	4.43	IV	III
Fossati et al. (35)	Sexual abuse, age 0–5 years	98/191	3	OR	3.22 (1.23, 8.46)	0.017	Yes	0.0	No/NP	3.22	IV	IV
Fossati et al. (35)	Sexual abuse, by father	252/454	6	OR	2.26 (1.00, 5.09)	0.050	Yes	16.4	Yes/NP	2.26	IV	IV
Fossati et al. (35)	Sexual abuse, by siblings	252/454	6	OR	3.04 (1.15, 8.02)	0.024	Yes	16.0	No/no	3.04	IV	IV
Fossati et al. (35)	Sexual abuse, by other relatives	252/454	6	OR	3.01 (1.28, 7.04)	0.011	Yes	11.6	No/no	3.01	IV	IV
Fossati et al. (35)	Sexual abuse, by non-relatives	252/454	6	OR	3.16 (1.93, 5.18)	1.0 × 10^−5^	No	0.0	No/NP	3.16	IV	III
Fossati et al. (35)	Sexual abuse, by any relative	62/91	2	OR	12.65 (2.77, 20.28)	0.001	NA	0.00	NA/no	12.65	IV	IV
Fossati et al. (35)	Sexual abuse, penetration	84/132	2	OR	5.57 (1.56, 19.80)	0.008	NA	0.0	NA/NP	5.57	IV	IV
Porter et al. (36)	Any childhood adversity (clinical population)	13,498/42,490	140	OR	2.64	0.008	Yes	85.1	Yes/yes	2.64	IV	IV
Porter et al. (36)	Any childhood adversity (clinical population)	5,054/11,785	45	OR	4.26	0.001	Yes	82.9	Yes/NP	4.26	IV	IV
Porter et al. (36)	Physical abuse (other personality disorders)	2,420/13,138	32	OR	2.51	0.008	No	55.1	No/NP	2.51	IV	IV
Porter et al. (36)	Physical abuse (psychosis)	780/2,078	16	OR	2.68	2.19 × 10^−9^	No	38.0	No/no	2.68	IV	IV
**Dependent personality disorder**												
Kane and Bornstein (37)	Any childhood maltreatment	NA/178,670	22	d	0.30 (0.21, 0.38)	1.0 × 10^−11^	No	56.0	No	1.71	IV	II
Kane and Bornstein (37)	Any childhood abuse (including psychiatric disorders)	NA/4,542	12	d	0.42 (0.28, 0.56)	3.6 × 10^−9^	No	57.1	Yes	2.14	IV	II
Kane and Bornstein (37)	Any childhood abuse (community only)	3,800/152,249	10	d	0.18 (0.01, 0.35)	0.040	Yes	46.7	No	1.38	IV	IV
Kane and Bornstein (37)	Physical abuse	6,309/36,023	5	d	0.29 (0.03, 0.56)	0.031	Yes	65.5	No	1.69	IV	IV
Kane and Bornstein (37)	Sexual abuse	3,854/35,623	7	d	0.42 (0.56, 0.68)	0.002	Yes	72.2	No	2.14	IV	IV
Kane and Bornstein (37)	Neglect	3,457/35,948	4	d	0.32 (0.09, 0.56)	0.007	Yes	56.8	No	1.78	IV	IV
Kane and Bornstein (37)	Any sexual/physical childhood abuse	111/1,066	4	d	0.43 (0.20, 0.20)	1.6 × 10^−4^	Yes	0.0	Yes	2.18	IV	III

*d, Cohen's d; OR, odds ratio; PI, prediction interval; I^2^, heterogeneity; eOR, equivalent OR; NA, not applicable; NP, not permitted*.

*Level of evidence I, convincing evidence; II, highly suggestive evidence; III, suggestive evidence; IV, weak evidence*.

**Table 4:** Non-significant (*p* > 0.05) risk/protective factors for personality disorders.

**Reference**	**Risk factor**	**Total number of cases/total population**	**Number of primary studies**	**Effect size metric**	**Random effects summary effect size (95% CI)**	***P* random**	**95% PI (including null value)**	***I^**2**^* %**	**Small-study effects/excess statistical significance**	**eOR**	**Level of evidence**
**Antisocial personality disorder**											
Nottell (39)	Genetics	45/316	4	OR	2.55 (0.95, 6.82)	0.063	Yes	79.3	Yes/yes	2.55	NS
Nottell (39)	Any childhood adversity	581/26,693	17	OR	1.69 (0.93, 3.07)	0.082	Yes	83.2	No/no	1.69	NS
Nottell (39)	Physical abuse	197/755	3	OR	1.32 (0.53, 2.61)	0.543	Yes	66.5	No/NP	1.32	NS
Nottell (39)	Separation and loss	54/459	3	OR	19.40 (0.73, 515.84)	0.076	Yes	85.3	No/NP	19.40	NS
Nottell (39)	Family socioeconomic status	79/24,316	3	OR	1.07 (0.44, 2.59)	0.881	Yes	62.1	Yes/no	1.07	NS
Nottell (39)	Family dysfunction	127/561	4	OR	1.49 (0.54, 4.11)	0.444	Yes	78.5	No/NP	1.49	NS
Nottell (39)	Neglect in childhood	186/1,129	3	OR	1.40 (0.73, 2.68)	0.308	Yes	66.3	Yes/yes	1.40	NS
Nottell (39)	Inconsistent discipline	113/430	2	OR	1.13 (0.39, 3.22)	0.822	NA	76.7	NA/yes	1.13	NS
**Borderline personality disorder**											
Winsper et al. (38)	Parental conflict	104/6,148	3	d	0.38 (−0.06, 0.82)	0.092	Yes	48.8	Yes/NP	1.99	NS
Fossati et al. (35)	Sexual abuse, (age 7–12 years)	89/196	3	OR	2.99 (0.86, 10.40)	0.085	Yes	70.2	No/NP	2.99	NS
Fossati et al. (35)	Sexual abuse, (age 13–18 years)	88/196	3	OR	3.03 (0.68, 13.47)	0.144	Yes	57.4	No/NP	3.03	NS
Fossati et al. (35)	Sexual abuse, by mother	252/454	6	OR	2.16 (0.62, 7.54)	0.224	Yes	0.0	Yes/NP	2.16	NS
Fossati et al. (35)	Sexual abuse, with use of force	84/135	2	OR	2.37 (0.76, 7.37)	0.135	NA	12.5	NA/NP	2.37	NS
Fossati et al. (35)	Sexual abuse, with disclosure	84/132	2	OR	0.86 (0.37, 0.37)	0.729	NA	0.0	NA/NP	0.86	NS
Fossati et al. (35)	Sexual abuse, with help	84/132	2	OR	1.08 (0.37, 3.17)	0.882	NA	0.0	NA/NP	1.08	NS
Fossati et al. (35)	Sexual abuse, fondling	84/132	2	OR	0.46 (0.12, 1.76)	0.259	NA	0.0	NA/NP	0.46	NS
Fossati et al. (35)	Sexual abuse, genital fondling	84/132	2	OR	2.05 (0.90, 4.61)	0.084	NA	0.0	NA/NP	2.05	NS
Fossati et al. (35)	Sexual abuse, oral sex	84/132	2	OR	1.85 (0.54, 6.32)	0.324	NA	0.1	NA/NP	1.85	NS
Fossati et al. (35)	Sexual abuse, single incident	333/528	7	OR	1.08 (0.60, 1.95)	0.803	Yes	0.0	Yes/NP	1.08	NS
Fossati et al. (35)	Sexual abuse, number of abusers	139/306	3	OR	2.05 (0.80, 5.21)	0.134	Yes	58.4	No/NP	2.05	NS
**Dependent personality disorder**											
Kane and Bornstein (37)	Emotional abuse	11,472/70,124	3	d	0.25 (−0.14, 0.63)	0.207	Yes	68.7	No/NP	2.05	NS

*d, Cohen's d; OR, odds ratio; PI, prediction interval; I^2^, heterogeneity; eOR, equivalent OR; NA, not applicable; NP, not permitted; NS, non-significant*.

### Quality Assessment of Included Articles

Based on the AMSTAR2 assessment, two meta-analyses (40%) met the moderate quality level and three (60%) were of low quality (Table 2).

### Summary of Associations

Thirty-five of the 56 analyzed associations (62.5%) presented a statistically significant effect (p < 0.05) under the random-effects model, but only 12 (21.4%) reached *p* < 10^−6^. Twenty-five associations (44.6%) presented a large heterogeneity (*I*^2^ > 50%), while only for 13 associations (23.2%) the 95% prediction interval did not include the null. In addition, the evidence for small-study effects and excess significance bias was noted for 20 (35.7%) and 16 (28.5%) associations, respectively.

### Associations for Antisocial Personality Disorder

A total of 14 of the 56 associations examined associations for antisocial personality disorder. Six of those presented a nominally statistically significant effect (*p* ≤ 0.05) and met the class IV evidence criteria while only 1 association reached *p* < 10^−6^. None of those associations were supported by class I, II, and III evidence. After excluding the criterion of 1,000 cases, one factor, namely, disorders/pathology during childhood, was upgraded to the class II evidence while two others, namely, psychobiological factor and behavioral factor, were upgraded to the class III evidence (Tables 3, 4).

### Associations for Borderline Personality Disorder

A total of 22 of the 56 associations examined associations for borderline personality disorder. Twenty-two of those presented a nominally statistically significant effect (*p* ≤ 0.05), while nine of those associations reached *p* < 10^−6^. None of those associations were supported by class I or III evidence (Table 3). Six associations were supported by class II evidence (Table 3) involving childhood emotional abuse, childhood emotional neglect, childhood any adversities, childhood physical abuse, childhood sexual abuse, and childhood physical neglect. Sixteen other associations were supported by class IV evidence (Table 3) and 12 were non-significant (Table 4). After excluding the criterion of 1,000 cases, all factors with class II evidence remained at the same level while two others, namely, sexual abuse overall and sexual abuse by non-relatives, were upgraded to the class III evidence.

### Associations for Dependent Personality Disorder

A total of 8 of the 56 associations examined associations for dependent personality disorder. Seven of those presented a nominally statistically significant effect (*p* ≤ 0.05) and met the class IV evidence criteria while only two associations reached *p* < 10^−6^. None of those associations were supported by class I, II, and III evidence. After excluding the criterion of 1,000 cases, two factors, namely, any childhood maltreatment and any childhood abuse in other clinical populations, were upgraded to the class II evidence, while another one, namely, any childhood abuse vs. no childhood abuse, was upgraded to the class III evidence (Tables 3, 4).

## Discussion

This is the first umbrella review pooling data from five meta-analyses on risk factors for personality disorders. Findings show that out of 56 associations between 26 potential environmental factors and antisocial, dependent, borderline personality disorder, despite 35 (62.5%) of the associations were nominally significant, only 6 (8.92%) associations met class II evidence for borderline personality disorder, involving childhood emotional abuse, childhood emotional neglect, childhood any adversities, childhood physical abuse, childhood sexual abuse, and childhood physical neglect. All other significant associations were classified as weak (class IV evidence).

These results likely reflect the epidemiological distribution of borderline personality disorders, which represents the most common personality disorders both in clinical populations (36) and in the young general population, with a lifetime prevalence cumulating to about 10% in university students (40). The relatively high prevalence of this condition is likely to have facilitated etiopathological research in this field and, consequently, accumulation of established evidence, reviewed in the current study. At the same time, it is also the most reliable diagnosis within personality disorders, with superior diagnostic reliability (Kappa 0.54) compared with other personality disorders, and similar diagnostic reliability to that observed in bipolar I disorders (0.56) or schizophrenia (0.46) (41). Given the severe individual and societal burden and impact of borderline personality disorder and the limited effect of psychological interventions (42), knowledge into risk factors associated with this condition may advance clinical care. The findings that childhood emotional abuse, emotional/physical neglect, physical/sexual abuse, and adversities in general emerge as robust risk factor for this condition align with multiple salient clinical features such as affect instability, emotion regulation difficulties, and maladaptive coping strategies including substance misuse and frequent self-harm (43, 44). Indeed, it has been suggested that several core experiences of borderline personality disorders may be understood as complex post-traumatic stress disorders (45). The current finding that individuals with a diagnosis of borderline personality disorders are consistently more likely to report childhood adversity than non-clinical controls is consistent with this strong clinical narrative linking childhood adversity and this condition, and show that evidence survives several stringent additional criteria making it into class II. Furthermore, the magnitude of these associations appeared as very large, with ORs superior to 5 in all cases: emotional abuse, 28.15; emotional neglect, OR 22.86; adversities, OR 14.32; physical abuse, OR 9.30; sexual abuse, OR 7.95; physical neglect, OR 5.73. Although it is important to note that these ORs relate to case–control studies not surviving prospective analyses, their large magnitude holds clinical relevance. For example, these findings indicate that childhood trauma should be systematically ascertained during the diagnostic assessment of suspected cases or during their initial clinical management. Notably, these ORs were not controlled against each other, albeit being likely correlated. The next generation of research should then develop a multivariable assessment interview to collect these multiple exposures in the same individuals assessed for a potential borderline personality disorder, thus allowing multivariable association analyses, yet accounting for multicollinearity of different risk factors. Another future development of research may involve exploring the transdiagnosticity of childhood trauma as potential risk factor for other mental disorders such as eating disorders, depressive disorders, anxiety disorders, and even psychotic disorders. These considerations are particularly relevant in the context of potential screening (46) and preventive interventions (47, 48) and mental health promotion initiatives (49, 50), because it could potentially be possible to target multiple outcomes/mental disorders by reducing childhood trauma.

Interestingly, childhood trauma exposure has been linked with neurobiological modifications in personality disorders, particularly borderline personality disorder. For example, variations in volumes of the main brain regions involved in BPD (especially amygdala and hippocampus) have been associated to adverse childhood experiences and trauma exposure (51–54). Also, there is evidence of an association between childhood trauma and alterations of the cortisol circadian rhythm and levels, indicating a deregulation of the HPA axis responsiveness, which could also affect hippocampal volumes (51, 55, 56). Yet, such alterations are not exclusively present in BPD, as well as childhood traumatic events are risk factors for other mental disorders (57), including dependent personality disorder. Hence, the specific component justifying such a high association between BPD and childhood adversities remains unknown.

The present work also has several limitations. First, none of the findings met class I evidence. However, this is due to the available evidence. Indeed, findings inform the field that more cohort studies assessing multidimensional risk factors for personality disorders are needed. Similarly, the lack of evidence on protective factors is also due to lack of eligible meta-analyses reporting on protective factors. Second, compared with umbrella reviews assessing credibility of evidence on risk factors for other mental or physical disorders (14–17, 58, 59), we included a limited number of meta-analyses. Again, this indicates that more research efforts should focus on this clinically relevant field. Third, despite the large ORs, carefully designed longitudinal research, including examination of dose–response relationships, is required before definitive conclusions can be drawn regarding any causal role played by childhood adversity in the development of borderline personality disorders.

In conclusion, this umbrella review shows that risk factors for borderline personality disorder occur during childhood, and primary prevention strategies should encompass a multidisciplinary mental health promotion activity going beyond health professionals, to protect children from any maltreatment, neglect, or abuse. Risk factors for other personality disorders have been poorly identified so far, and more longitudinal studies should be conducted to inform prevention strategies.

## Data Availability Statement

The raw data supporting the conclusions of this article will be made available by the authors, upon request.

## Author Contributions

AFC, MS, PF-P, and ED designed the study. GC, PK, ED, and MS, extracted the data. ED run the analyses. MS and ED drafted the first version of the manuscript. All authors contributed to the manuscript, revised it, and approved the final version and protocol.

## Conflict of Interest

MS received fee/honoraria from Angelini, Lundbeck. SC declares honoraria and reimbursement for travel and accommodation expenses for lectures from the following non-profit associations: Association for Child and Adolescent Central Health (ACAMH), Canadian ADHD Alliance Resource (CADDRA), British Association of Pharmacology (BAP), and from Healthcare Convention for educational activity on ADHD. PF-P has received research fees from Lundbeck and honoraria from Lundbeck, Angelini, Menarini, and Boehringer Ingelheim outside the current study. The remaining authors declare that the research was conducted in the absence of any commercial or financial relationships that could be construed as a potential conflict of interest.

## Publisher's Note

All claims expressed in this article are solely those of the authors and do not necessarily represent those of their affiliated organizations, or those of the publisher, the editors and the reviewers. Any product that may be evaluated in this article, or claim that may be made by its manufacturer, is not guaranteed or endorsed by the publisher.
