# Supplementary material for: Risk and Protective Factors for Personality Disorders: An Umbrella Review of Published Meta-Analyses of Case–Control and Cohort Studies
Source: Front Psychiatry. 2021 Sep 6;12:679379. doi: 10.3389/fpsyt.2021.679379 (PMC8450571; doi:10.3389/fpsyt.2021.679379)
Supplement: Supplementary file 1 [file Data_Sheet_1.docx]

**Risk and protective factors for personality disorders: an umbrella review of published meta-analyses of case-control and cohort studies**

Marco Solmi,^§^ Elena Dragioti, Giovanni Croatto, Joaquim Radua, Stefan Borgwardt, Andre F. Carvalho, Jacopo Demurtas, Anna Mosina, Peter Kurotschka, Trevor Thompson, Samuele Cortese, Jae Il Shin, Paolo Fusar-Poli

Corresponding author

Dr. Marco Solmi

Neurosciences Department, University of Padua, Padua, Italy

Via Giustiniani, 5 – 35100 – Padua, Italy

+39-0498213831 - [Marco.solmi83@gmail.com](mailto:Marco.solmi83@gmail.com)

§ joint first authors

# co-corresponding author

*Keywords: umbrella review; personality disorder; risk factor; prevention; systematic review; meta-analysis; mental health; psychiatry*

**Index**

eTable 1. PRISMA checklist

eTable 2. MOOSE checklist

eTable 3. The list of excluded meta-analyses by full text screening with exclusion reason

**eTable 1. PRISMA checklist**(1)

| **Section/topic** | **#** | **Checklist item** | **Reported on page #** | |
| --- | --- | --- | --- | --- |
| **TITLE** | | | |  |
| Title | 1 | Identify the report as a systematic review, meta-analysis, or both. | 1 | |
| **ABSTRACT** | | | |  |
| Structured summary | 2 | Provide a structured summary including, as applicable: background; objectives; data sources; study eligibility criteria, participants, and interventions; study appraisal and synthesis methods; results; limitations; conclusions and implications of key findings; systematic review registration number. | 2 | |
| **INTRODUCTION** | | | |  |
| Rationale | 3 | Describe the rationale for the review in the context of what is already known. | 3 | |
| Objectives | 4 | Provide an explicit statement of questions being addressed with reference to participants, interventions, comparisons, outcomes, and study design (PICOS). | 4 | |
| **METHODS** | | | |  |
| Protocol and registration | 5 | Indicate if a review protocol exists, if and where it can be accessed (e.g., Web address), and, if available, provide registration information including registration number. | 4 | |
| Eligibility criteria | 6 | Specify study characteristics (e.g., PICOS, length of follow-up) and report characteristics (e.g., years considered, language, publication status) used as criteria for eligibility, giving rationale. | 4 | |
| Information sources | 7 | Describe all information sources (e.g., databases with dates of coverage, contact with study authors to identify additional studies) in the search and date last searched. | 4 | |
| Search | 8 | Present full electronic search strategy for at least one database, including any limits used, such that it could be repeated. | 4 | |
| Study selection | 9 | State the process for selecting studies (i.e., screening, eligibility, included in systematic review, and, if applicable, included in the meta-analysis). | 4 | |
| Data collection process | 10 | Describe method of data extraction from reports (e.g., piloted forms, independently, in duplicate) and any processes for obtaining and confirming data from investigators. | 4 | |
| Data items | 11 | List and define all variables for which data were sought (e.g., PICOS, funding sources) and any assumptions and simplifications made. | 5 | |
| Risk of bias in individual studies | 12 | Describe methods used for assessing risk of bias of individual studies (including specification of whether this was done at the study or outcome level), and how this information is to be used in any data synthesis. | 5 | |
| Summary measures | 13 | State the principal summary measures (e.g., risk ratio, difference in means). | 5 | |
| Synthesis of results | 14 | Describe the methods of handling data and combining results of studies, if done, including measures of consistency (e.g., I^2^) for each meta-analysis. | 5 | |
| Risk of bias across studies | 15 | Specify any assessment of risk of bias that may affect the cumulative evidence (e.g., publication bias, selective reporting within studies). | 5 | |
| Additional analyses | 16 | Describe methods of additional analyses (e.g., sensitivity or subgroup analyses, meta-regression), if done, indicating which were pre-specified. | 6 | |
| **RESULTS** | | |  | |
| Study selection | 17 | Give numbers of studies screened, assessed for eligibility, and included in the review, with reasons for exclusions at each stage, ideally with a flow diagram. | 6 | |
| Study characteristics | 18 | For each study, present characteristics for which data were extracted (e.g., study size, PICOS, follow-up period) and provide the citations. | 6 | |
| Risk of bias within studies | 19 | Present data on risk of bias of each study and, if available, any outcome level assessment (see item 12). | 7 | |
| Results of individual studies | 20 | For all outcomes considered (benefits or harms), present, for each study: (a) simple summary data for each intervention group (b) effect estimates and confidence intervals, ideally with a forest plot. | 6 | |
| Synthesis of results | 21 | Present results of each meta-analysis done, including confidence intervals and measures of consistency. | 6 | |
| Risk of bias across studies | 22 | Present results of any assessment of risk of bias across studies (see Item 15). | 7 | |
| Additional analysis | 23 | Give results of additional analyses, if done (e.g., sensitivity or subgroup analyses, meta-regression [see Item 16]). | 6-7 | |
| **DISCUSSION** | | |  | |
| Summary of evidence | 24 | Summarize the main findings including the strength of evidence for each main outcome; consider their relevance to key groups (e.g., healthcare providers, users, and policy makers). | 7-8 | |
| Limitations | 25 | Discuss limitations at study and outcome level (e.g., risk of bias), and at review-level (e.g., incomplete retrieval of identified research, reporting bias). | 8 | |
| Conclusions | 26 | Provide a general interpretation of the results in the context of other evidence, and implications for future research. | 8-9 | |
| **FUNDING** | | |  | |
| Funding | 27 | Describe sources of funding for the systematic review and other support (e.g., supply of data); role of funders for the systematic review. | 9 | |

*^1^From:*  Moher D, Liberati A, Tetzlaff J, Altman DG, The PRISMA Group (2009). Preferred Reporting Items for Systematic Reviews and Meta-Analyses: The PRISMA Statement. PLoS Med 6(7): e1000097. doi:10.1371/journal.pmed1000097

eTable 2. MOOSE Checklist for Meta-analyses of Observational Studies**(2)**

| **Item No** | **Recommendation** | **Reported**  **on Page No** |
| --- | --- | --- |
| Reporting of background should include | | |
| 1 | Problem definition | 3 |
| 2 | Hypothesis statement | 3 |
| 3 | Description of study outcome(s) | 3 |
| 4 | Type of exposure or intervention used | 3 |
| 5 | Type of study designs used | 3 |
| 6 | Study population | 4 |
| Reporting of search strategy should include | | |
| 7 | Qualifications of searchers (eg, librarians and investigators) | 4 |
| 8 | Search strategy, including time period included in the synthesis and key words | 4 |
| 9 | Effort to include all available studies, including contact with authors | 5 |
| 10 | Databases and registries searched | 4 |
| 11 | Search software used, name and version, including special features used (eg, explosion) | 4 |
| 12 | Use of hand searching (eg, reference lists of obtained articles) | 4 |
| 13 | List of citations located and those excluded, including justification | 6, Table 2, eTable 3 |
| 14 | Method of addressing articles published in languages other than English | 4 |
| 15 | Method of handling abstracts and unpublished studies | 5 |
| 16 | Description of any contact with authors | 5 |
| Reporting of methods should include | | |
| 17 | Description of relevance or appropriateness of studies assembled for assessing the hypothesis to be tested | 3-4 |
| 18 | Rationale for the selection and coding of data (eg, sound clinical principles or convenience) | 4-5 |
| 19 | Documentation of how data were classified and coded (eg, multiple raters, blinding and interrater reliability) | 5 |
| 20 | Assessment of confounding (eg, comparability of cases and controls in studies where appropriate) | 5 |
| 21 | Assessment of study quality, including blinding of quality assessors, stratification or regression on possible predictors of study results | 4-5 |
| 22 | Assessment of heterogeneity | 5 |
| 23 | Description of statistical methods (eg, complete description of fixed or random effects models, justification of whether the chosen models account for predictors of study results, dose-response models, or cumulative meta-analysis) in sufficient detail to be replicated | 5, Table 1 |
| 24 | Provision of appropriate tables and graphics | Table 1, 2, 3,4 Figure 1, eTable 1, 2, 3 |
| Reporting of results should include | | |
| 25 | Graphic summarizing individual study estimates and overall estimate | NA |
| 26 | Table giving descriptive information for each study included | Table 3,4 |
| 27 | Results of sensitivity testing (eg, subgroup analysis) | Table 3,4 |
| 28 | Indication of statistical uncertainty of findings | 6, 7, 8, Table 3,4 |

| **Item No** | **Recommendation** | **Reported**  **on Page No** |
| --- | --- | --- |
| Reporting of discussion should include | | |
| 29 | Quantitative assessment of bias (eg, publication bias) | 7, 8, 9, Table 3,4 |
| 30 | Justification for exclusion (eg, exclusion of non-English language citations) | eTable 3 |
| 31 | Assessment of quality of included studies | 7-8, Table 3,4 |
| Reporting of conclusions should include | | |
| 32 | Consideration of alternative explanations for observed results | 8 |
| 33 | Generalization of the conclusions (ie, appropriate for the data presented and within the domain of the literature review) | 8 |
| 34 | Guidelines for future research | 9 |
| 35 | Disclosure of funding source | 9 |

## **eTable 3 The list of excluded articles by full text screening with exclusion reason**

| **Reference** | **Reason for exclusion** |
| --- | --- |
| Dinsdale, 2016 (3) | Cross-sectional studies |
| Foxhall, 2019 (4) | Cross-sectional studies |
| Gao, 2017 (5) | Cross-sectional studies |
| Derks, 2017 (6) | Cross-sectional studies |
| Pyhala, 2017(7) | No DSM/ICD personality disorder |
| Burt, 2009 (8) | No DSM/ICD personality disorder |
| Jezior, 2018 (9) | No DSM/ICD personality disorder |
| Keinanen, 2012(10) | No meta-analysis |
| Laulik, 2013 (11) | No meta-analysis |
| Petfield, 2015(12) | No meta-analysis |
| Ibrahim, 2018(13) | No meta-analysis |
| Eyden, 2016(14) | No meta-analysis |
| Stepp, 2016 (15) | No meta-analysis |
| Boucher, 2017 (16) | No meta-analysis |
| Fazel, 2008 (17) | No risk or protective factor |
| Steele, 2019 (18) | Overview of reviews |

Supplementary References

1. Moher D, Liberati A, Tetzlaff J, Altman DG. Preferred reporting items for systematic reviews and meta-analyses: the PRISMA statement. *J Clin Epidemiol* (2009) **62**:1006–1012. doi:10.1016/j.jclinepi.2009.06.005

2. Stroup DF, Berlin JA, Morton SC, Olkin I, Williamson GD, Rennie D, Moher D, Becker BJ, Sipe TA, Thacker SB. Meta-analysis of observational studies in epidemiology: A proposal for reporting. *J Am Med Assoc* (2000) **283**:2008–2012. doi:10.1001/jama.283.15.2008

3. Dinsdale N, Mokkonen M, Crespi B. The ‘extreme female brain’: increased cognitive empathy as a dimension of psychopathology. *Evol Hum Behav* (2016) **37**:323–336. doi:https://doi.org/10.1016/j.evolhumbehav.2016.02.003

4. Foxhall M, Hamilton-Giachritsis C, Button K. The link between rejection sensitivity and borderline personality disorder: A systematic review and meta-analysis. *Br J Clin Psychol* (2019) **58**:289–326. doi:10.1111/bjc.12216

5. Gao S, Assink M, Cipriani A, Lin K. Associations between rejection sensitivity and mental health outcomes: A meta-analytic review. *Clin Psychol Rev* (2017) **57**:59–74. doi:https://doi.org/10.1016/j.cpr.2017.08.007

6. Derks YPMJ, Westerhof GJ, Bohlmeijer ET. A meta-analysis on the association between emotional awareness and borderline personality pathology. *J Pers Disord* (2017) **31**:362–384. doi:10.1521/pedi_2016_30_257

7. Pyhälä R, Wolford E, Kautiainen H, Andersson S, Bartmann P, Baumann N, Brubakk A-M, Evensen KAI, Hovi P, Kajantie E, et al. Self-Reported Mental Health Problems Among Adults Born Preterm: A Meta-analysis. *Pediatrics* (2017) **139**:e20162690. doi:10.1542/peds.2016-2690

8. Burt SA. Are there meaningful etiological differences within antisocial behavior? Results of a meta-analysis. *Clin Psychol Rev* (2009) **29**:163–178. doi:https://doi.org/10.1016/j.cpr.2008.12.004

9. Oppositional Defiant Disorder: Meta-Analysis, Predictive Validity, and Mediation of Psychopathology Outcomes. Available at: https://escholarship.org/uc/item/9f009307 [Accessed September 15, 2020]

10. Keinänen M, Johnson J, Richards E, Courtney E. A systematic review of the evidence-based psychosocial risk factors for understanding of borderline personality disorder. *Psychoanal Psychother* (2012) **26**:65–91. doi:10.1080/02668734.2011.652659

11. Laulik S, Chou S, Browne KD, Allam J. The link between personality disorder and parenting behaviors: A systematic review. *Aggress Violent Behav* (2013) **18**:644–655. doi:10.1016/j.avb.2013.07.017

12. Petfield L, Startup H, Droscher H, Cartwright-Hatton S. Parenting in mothers with borderline personality disorder and impact on child outcomes. *Evid Based Ment Heal* (2015) **18**:67 LP – 75. doi:10.1136/eb-2015-102163

13. Ibrahim J, Cosgrave N, Woolgar M. Childhood maltreatment and its link to borderline personality disorder features in children: A systematic review approach. *Clin Child Psychol Psychiatry* (2018) **23**:57–76. doi:10.1177/1359104517712778

14. Eyden J, Winsper C, Wolke D, Broome MR, MacCallum F. A systematic review of the parenting and outcomes experienced by offspring of mothers with borderline personality pathology: Potential mechanisms and clinical implications. *Clin Psychol Rev* (2016) **47**:85–105. doi:https://doi.org/10.1016/j.cpr.2016.04.002

15. Stepp SD, Lazarus SA, Byrd AL. A systematic review of risk factors prospectively associated with borderline personality disorder: Taking stock and moving forward. *Personal Disord* (2016) **7**:316–323. doi:10.1037/per0000186

16. Boucher M-È, Pugliese J, Allard-Chapais C, Lecours S, Ahoundova L, Chouinard R, Gaham S. Parent–child relationship associated with the development of borderline personality disorder: A systematic review. *Personal Ment Health* (2017) **11**:229–255. doi:10.1002/pmh.1385

17. Fazel S, Khosla V, Doll H, Geddes J. The Prevalence of Mental Disorders among the Homeless in Western Countries: Systematic Review and Meta-Regression Analysis. *PLOS Med* (2008) **5**:e225. Available at: https://doi.org/10.1371/journal.pmed.0050225

18. Steele KR, Townsend ML, Grenyer BFS. Parenting and personality disorder: An overview and meta-synthesis of systematic reviews. *PLoS One* (2019) **14**:e0223038–e0223038. doi:10.1371/journal.pone.0223038
